# Influence of assessment site in measuring transcutaneous bilirubin

**DOI:** 10.1590/S1679-45082014AO2711

**Published:** 2014

**Authors:** Cristiane Maria da Conceição, Maria Fernanda Pellegrino da Silva Dornaus, Maria Aparecida Portella, Alice D'Agostini Deutsch, Celso Moura Rebello

**Affiliations:** 1 Hospital Israelita Albert Einstein, São Paulo, SP, Brazil

**Keywords:** Bilirubin, Jaundice, neonatal/diagnosis, Infant, newborn

## Abstract

**Objective:**

To investigate the influence of the site of measurement of transcutaneous bilirubin (forehead or sternum) in reproducibility of results as compared to plasma bilirubin.

**Methods:**

A cohort study including 58 term newborns with no hemolytic disease. Transcutaneous measurements were performed on the forehead (halfway between the headline and the glabella, from the left toward the right side, making consecutive determinations, one-centimeter apart) and the sternum (five measurements, from the suprasternal notch to the xiphoid process with consecutive determinations, one-centimeter apart) using Bilicheck^®^ (SpectRx Inc, Norcross, Georgia, USA). The correlation and agreement between both methods and plasma bilirubin were calculated.

**Results:**

There was a strong linear correlation between both determinations of serum bilirubin at the forehead and sternum (r=0.704; p<0.01 and r=0.653; p<0.01, respectively). There was correspondence of the mean values of transcutaneous bilirubin measured on the sternum (9.9±2.2mg/dL) compared to plasma levels (10.2±1.7mg/dL), but both differ from the values measured on the forehead (8.6±2.0mg/dL), p<0.05.

**Conclusion:**

In newborn term infants with no hemolytic disease, measuring of transcutaneous bilirubin on the sternum had higher accuracy as compared to serum bilirubin measurement on the forehead.

## RESUMO

**Objetivo:**

Verificar a influência do local da mensuração da bilirrubina transcutânea (fronte ou esterno) na reprodutibilidade dos resultados, quando comparada à bilirrubina plasmática.

**Métodos:**

Estudo tipo coorte incluindo 58 recém-nascidos a termo sem doença hemolítica. Foram realizadas determinações transcutâneas na fronte (a meia distância entre a raiz do couro cabeludo e a glabela, iniciando do lado esquerdo em direção ao direito, realizando determinações consecutivas, separadas por 1cm) e no esterno (cinco mensurações iniciando da fúrcula até o apêndice xifoide, com determinações consecutivas, separadas por 1cm), utilizando o equipamento Bilicheck^®^ (SpectRx Inc, Norcross, Geórgia, Estados Unidos). Foram calculadas as correlações e a concordância entre ambos os métodos, e a bilirrubina plasmática.

**Resultados:**

Foi encontrada forte correlação linear tanto entre a determinação na fronte quanto no esterno, com nível sérico de bilirrubina (r=0,704; p<0,01 e r=0,653; p<0,01, respectivamente). Houve correspondência dos valores médios de bilirrubina transcutânea aferidos no esterno (9,9±2,2mg/dL) com os valores plasmáticos (10,2±1,7mg/dL), porém ambos diferiram dos valores medidos na fronte (8,6±2,0mg/dL), com p<0,05.

**Conclusão:**

Em recém-nascidos a termo sem doença hemolítica, a mensuração da bilirrubina transcutânea realizada no esterno apresenta maior acurácia em relação à mensuração na fronte, quando comparada à bilirrubina sérica.

## INTRODUCTION

Most newborn (NB) infants develop jaundice in the first week of life. It occurs in up to 92% of term and late preterm NB infants.^(1)^ The occurrence of high serum bilirubin levels for prolonged time may permanently damage structures of the Central Nervous System, such as the globus pallidus, subthalamic nuclei, hippocampus, and oculomotor nucleus, among others, leading to kernicterus.^(2)^

The indication of phototherapy for treating neonatal jaundice will depend on serum bilirubin levels, presence of blood incompatibility, weight, and chronological and gestational ages, in addition to associated comorbidities.^(3)^ Hence the American Academy of Pediatrics recommends that every NB have its bilirubin level measured before hospital discharge and this measurement should be repeated on the first days after discharge.^(4)^

Invasive bilirubin dosing demands drawing blood, with many inconveniences, such as technical difficulties in venous puncture, delay to obtain results, discomfort caused by pain^(5,6)^, and parental stress^(7)^, so it is important to minimize not only the amount of blood the NB loses in blood draws, but also to reduce to number of draws to the minimum possible.^(8)^

In that sense, in the early 1980's non-invasive (transcutaneous) techniques were developed to measure bilirubin and minimize the inconvenience of blood draws. The first equipment developed only correlated the intensity of yellow skin color with bilirubinemia, suffering the interference of many factors, like the amount of melanin, hemoglobin and connective tissue.^(5)^

In the past few years, a new generation of devices for the transcutaneous measurement of bilirubin has been produced^(6)^. They differ from previous models for being based on microspectrometry, which enables determining the optical density of bilirubin, hemoglobin and melanin in the subcutaneous layer of the NB infant skin. Excluding the factors that interfere in the determination of bilirubin leads to measuring its optical density in the subcutaneous capillaries and tissues with greater accuracy,^(6)^ thus enabling the replacement of plasma measurements for transcutaneous measurements.^(9)^ This technique has been studied in our context, and a good correlation between transcutaneous and serum bilirubin^(10)^ has been found even in a multiracial population.^(11)^ Recently, a meta-analysis gathered the results of 21 studies comparing transcutaneous bilirubin to serum bilirubin in preterm infants, confirming the accuracy of this technique also in this NB population.^(12)^

On the other hand, bilirubin measurement may suffer the influence of the site of measurement: forehead or sternum.^(13)^ The literature presents conflicting results, and it was demonstrated in term NB that transcutaneous measurements on the forehead and sternum are equivalent.^(10,14–16)^ Moreover, sternal measurement results in higher bilirubin levels than on the forehead.^(17–19)^

## OBJECTIVE

To verify the influence of the measurement site of transcutaneous bilirubin, forehead or sternum, in the reproducibility of results, as compared to plasma bilirubin.

## METHODS

After approval by the Research Ethics Committee (REF CEP/Einstein 08/896), a prospective cohort study was conducted including healthy term infants born at the *Hospital Israelita Albert Einstein*, a private tertiary-care hospital in the city of São Paulo (SP), in the period between April to September 2009. NB with gestational age ≥37 weeks with <72 hours of life were included. Newborn infants with hemolytic disease, with skin abnormalities or previous phototherapy treatment were excluded. Jaundice due to hemolytic disease was defined as that with early onset (in the first 48 hours of life), or with laboratory values that were incompatible with physiological jaundice (occurrence of reticulocytosis, positive Coombs or eluate tests). After obtaining the informed consent from parents or legal guardian, NB infants with clinical indication of plasma bilirubin measurement, according to the routine assessment by the neonatologist, had bilirubin measured transcutaneously too.

Immediately after the collection of plasma bilirubin, always the same researcher measured transcutaneous bilirubin on the forehead and on the sternum, using the Bilicheck^®^ device (SpectRx Inc, Norcross, Georgia, USA). The device was calibrated before each measurement, according to the manufacturer's instructions to ensure measurement accuracy.^(20)^ For each measurement, the device was positioned on the infant's skin, and five individual measurements in different points led to one result. On the forehead, the five measurements were taken halfway the hairline and glabella, starting on the left towards right side, one-centimeter apart. On the sternum, five measurements were taken, starting on the suprasternal notch to the xiphoid process, with consecutive one-centimeter apart determinations.

The statistical analysis was performed through the calculation of Pearson's correlation coefficient for both transcutaneous measurements compared to plasma measurements, and then Bland-Altman charts were plotted to assess agreement, and, finally, simple variance analysis (one-way ANOVA) to compare means, with Student-Newman-Keuls as a discriminatory post-test. The calculated size of the sample was 50 NB, considering the difference between means to be detected as 1.0mg/dL, with an expected standard deviation of 2.4mg/dL^(17)^, 0.80 power test, and 0.05 significance level. For the statistical analysis, Sigma Stat software, version 2.0 was used.

## RESULTS

**Figure 1:**
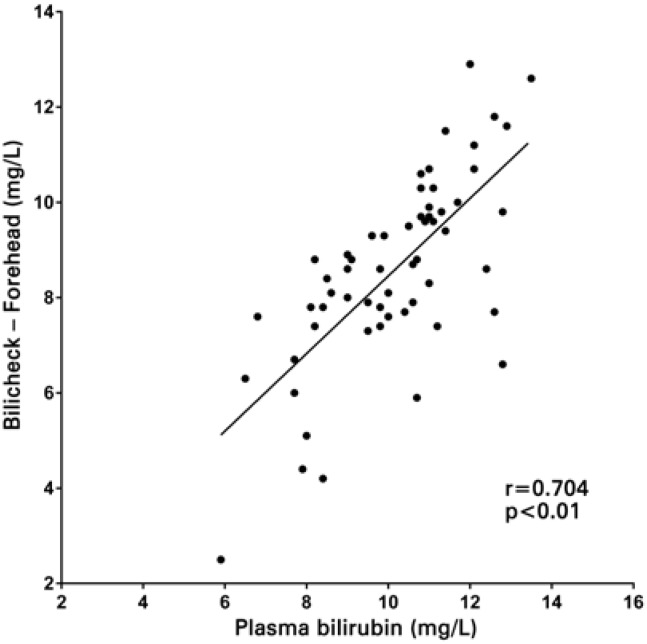
Linear correlation between transcutaneous measurement on the forehead and serum total bilirubin levels

**Figure 2:**
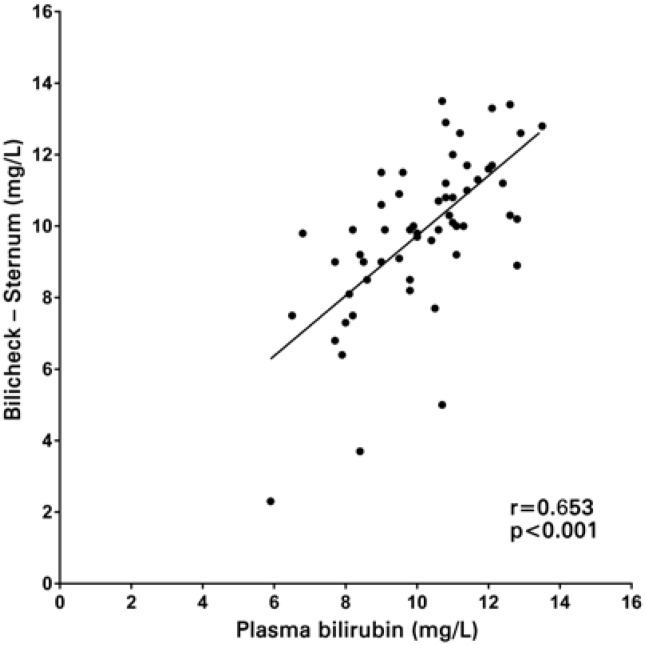
Linear correlation between transcutaneous measurement on the sternum and serum total bilirubin level

**Figure 3:**
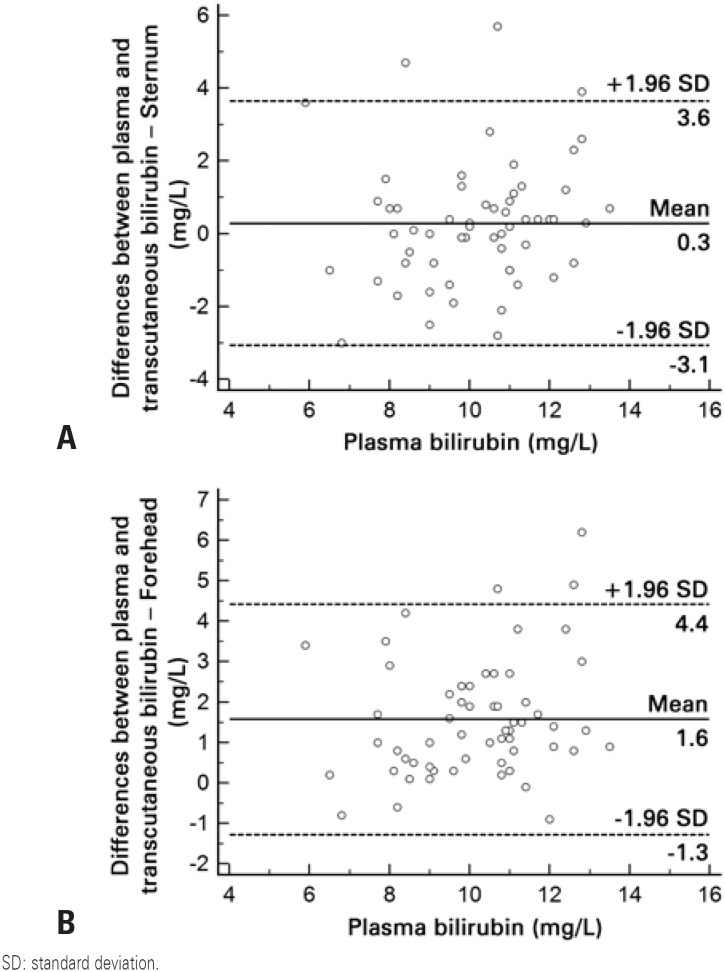
Bland-Altman charts plotted with the differences in the results between transcutaneous measurements on the sternum and (A), and transcutaneous measurements taken on the forehead and plasma bilirubin (B)

**Figure 4:**
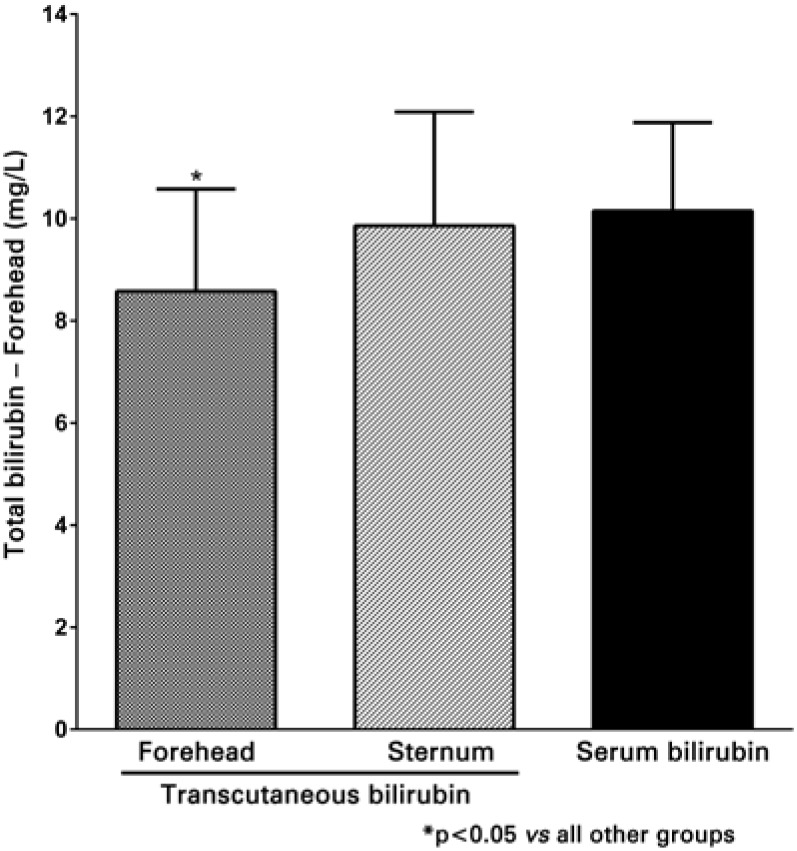
Bilirubin levels measured on the forehead, sternum and plasma. Values as mean±standard deviation (DP)

A total of 58 NB were studied, with birth weight of 3221±402g (mean±standard deviation) gestational age of 38.4±1.2 weeks, one-minute Apgar score of 9.0±0.3 and five-minute Apgar score of 10.0±0.0. All NB infants were Caucasian, and measurements were taken with 1.8±0.9 days of life. A total of 94.8% of the NB included were born from C-sections and 5.2% from vaginal deliveries. A good linear correlation was observed both between transcutaneous bilirubin measurements on the forehead and serum levels (r=0.704; p<0.01), as well as between transcutaneous measurements on the sternum and serum levels (r=0.653; p<0.01) (Figures 1 and 2). Differences in the results between transcutaneous measurements on the forehead and sternum and total serum bilirubin are shown in figures 3A and 3B. The mean difference between transcutaneous measurements from the sternum and plasma bilirubin was 0.3mg/dL, number below that found in the difference between measurements taken on the forehead and corresponding plasma bilirubin levels (1.6mg/dL). The comparison between the means of bilirubin values found on the forehead, sternum, as well as the corresponding serum bilirubin is shown in figure 4. There was a correspondence of the values measured on the sternum with plasma values, but both differed from the values measured on the forehead (p<0.05).

## DISCUSSION

This study had the objective of verifying in term NB with no hemolytic disease the influence of transcutaneous bilirubin measurement site in accuracy of results. The principal contribution of this study was to provide, with data obtained in our environment, information about an issue that the international literature shows non-homogeneous, and sometimes conflicting, results.

Accuracy in the measurement of transcutaneous bilirubin in relation to serum bilirubin has been recently demonstrated in a meta-analysis gathering data from 3527 patients published in 21 studies. In 16 of those studies, measurements were taken on the forehead, in 10 on the sternum, and in 3 on the abdomen.^(12)^ Our data demonstrate that measurements taken on the sternum have a good correlation with serum measurements, unlike measurements taken on the forehead. Possibly as a result of the continuous exposure to room light, bilirubin measurements in areas that are not covered by clothes, like the face, may present lower bilirubin values. Many authors tried to relate the measurement site of transcutaneous bilirubin (forehead, sternum, dorsum, knee, or foot) with accuracy of results,^(13)^ and the measurements taken on the forehead and sternum presented the best correlations with serum bilirubin. ^(6,17,21,22)^ Like the results of this study, Maisels et al. found a better correlation with serum bilirubin when transcutaneous measurements were taken on the sternum (r=0.953) as compared to measurements on the forehead (r=0,914).^(18)^ Similarly to our results, a revision published in 2009, including 13 studies addressing the influence of measurement site on the results of transcutaneous bilirubin, concluded that the sternum presents good correlation with serum bilirubin. However, in six studies of this revision, no differences were observed between measurements taken on the forehead and sternum, and, in two studies, measurements taken on the forehead were more reliable than those taken on the sternum.^(13)^

In another study, transcutaneous measurement of bilirubin taken on the forehead suffered the influence of crying, and the lowest values were found in the NB who were crying at the time of measurement.^(23)^ In our study, NB infants were not crying upon measurement.

In disagreement with this study, Bertini and Rubaltelli demonstrated that the precision of transcutaneous measurements, when taken on the forehead and sternum, are comparable, but sternum measurements are, in average, 0.8 to 0.9mg/dL higher.^(10)^ Likewise, the average of transcutaneous bilirubin measurements taken on the trunk was demonstrated to be 0.4mg/dL higher than serum measurements, whereas measurements on the forehead were 0.3mg/dL (5mol/L) smaller than serum measurements. The authors concluded that plasma measurements and transcutaneous measurements showed approximate values, but after hospital discharge, forehead measurements underestimated values by 5%. The authors recommend using trunk measurements for bilirubinemia.^(15)^

Similar results to this study were found in a group of 345 NB, in which a better correlation between blood bilirubin and transcutaneous measurements taken on the sternum than on the forehead were found.^(19)^ Even though accuracy is similar for measurements taken on the frontal region or on the sternum, correlation is greater on the latter, possibly due to the head's exposure to room light.^(24)^

Disagreeing with the findings of this study, in our environment, a group of 44 NB, with average gestational age of 35.1±3.4 weeks and average birth weight of 2151±889g, 73% of them Caucasian, was analyzed between the second and third days of life. The authors did not find differences between serum bilirubin levels and transcutaneous bilirubin levels measured on covered areas of the forehead and sternum 24 hours after the beginning of phototherapy.^(16)^

This study presents some weaknesses, including its relatively small sample formed exclusively by term Caucasian NB with no hemolytic disease. The inclusion of only Caucasian NB infants was unintentional, at the time of patient recruitment, which resulted in a sample that is different from that of the Brazilian population. But this fact does not compromise significantly our conclusions. It is important, however, to highlight that the influence of the measurement site of transcutaneous bilirubin (forehead or sternum) on serum bilirubin should be also assessed in preterm infants of other races and with hemolytic disease, before generalizing these conclusions to these groups.

## CONCLUSION

In Caucasian term NB without hemolytic disease, transcutaneous bilirubin measurement taken on the sternum presents greater accuracy than forehead measurements, when compared to serum bilirubin.
